# Recommendations for Updating Fever and Inflammation of Unknown Origin From a Modified Delphi Consensus Panel

**DOI:** 10.1093/ofid/ofae298

**Published:** 2024-06-10

**Authors:** William F Wright, Lauren Stelmash, Albrecht Betrains, Catharina M Mulders-Manders, Chantal P Rovers, Steven Vanderschueren, Paul G Auwaerter, International Fever and Inflammation of Unknown Origin Research Working Group, Cristian Baicus, Tehmina Bharucha, David Bor, Mile Bosilkovski, Michael Brown, Karen Carroll, Francesco Maria Fusco, Gavin Harris, Toshio Naito, Wim JG Oyen, Seve Pascal, Steven Rowe, Priscilla Rupali, Lynora Saxinger, Terasawa Teruhiko, Ercan Yenilmez, Thierry Zenone

**Affiliations:** Division of Infectious Diseases, Department of Medicine, Johns Hopkins University School of Medicine, Baltimore, Maryland, USA; Division of Infectious Diseases, Department of Medicine, Johns Hopkins Bayview Medical Center, Baltimore, Maryland, USA; General Internal Medicine department, University Hospitals Leuven, Leuven, Belgium; Division of Infectious Diseases, Department of Internal Medicine, Radboud University Medical Center, Nijmegen, the Netherlands; Division of Infectious Diseases, Department of Internal Medicine, Radboud University Medical Center, Nijmegen, the Netherlands; General Internal Medicine Department, Department of Microbiology, Immunology, and Transplantation, Laboratory of Clinical Infectious and Inflammatory Disorders, University Hospitals Leuven, KU Leuven, Leuven, Belgium; The Sherrilyn and Ken Fisher Center for Environmental Infectious Diseases, Johns Hopkins University School of Medicine, Baltimore, Maryland, USA

**Keywords:** fever, fever of unknown origin, inflammation of unknown origin, pyrexia, pyrexia of unknown origin

## Abstract

**Background:**

Fever of unknown origin (FUO) and inflammation of unknown origin (IUO) are syndromes commonly used as medical diagnoses. Since the existing literature has a mixture of diagnostic approaches, developing consensus-based recommendations would be helpful for clinicians, researchers, and patients.

**Methods:**

A modified Delphi process was performed from October 2022 to July 2023, involving 4 rounds of online surveys and 2 live video conferences. The panel comprised international experts recruited based on peer-reviewed published publications and studies.

**Results:**

Among 50 invited experts, 26 (52.0%) agreed to participate. Twenty-three panelists completed round 1 of the survey, 21 completed rounds 2 and 3, 20 completed round 4, and 7 participated in round 5 live video discussions. Of the participants, 18 (78.3%) were academic-based clinicians and researchers, 5 (21.7%) practiced in a community-based hospital, and 6 (26.1%) were female. Consensus was reached on 5 themes: (1) incorporating epidemiologic factors, such as geographic location and travel history; (2) updated criteria for classifying FUO or IUO; (3) initial evaluation approaches; (4) a classification system for diagnoses; and (5) recommendations for judicious limitation of empiric therapies. Experts strongly disagreed with using 2-deoxy-2-[^18^F] fluoro-D-glucose positron emission tomography/computed tomography as part of the diagnostic criteria for FUO. There were mixed opinions about the importance of the temperature measurement site, the 3-week minimum illness criterion, the need for a standard definition of relapsing fevers, and the use of similar evaluation strategies for FUO and IUO.

**Conclusions:**

These Delphi-generated consensus-based recommendations offer potential improvements compared with earlier definitions and a guide for clinical practice and future research.

Advances in clinical research and diagnostic testing methods over the last 3 decades suggest that an update for existing definitions and approaches for fever and inflammation of unknown origin is needed [1–4]. Classic fever of unknown origin (FUO) was first robustly studied in 1961, with subsequent modifications in definitions (Table 1) [1, 2]. These FUO definitions require an illness of ≥3 weeks duration and temperatures of ≥38.3°C (≥100.9°F) on several occasions [1, 2]. However, other criteria differ, such as which kind of initial testing is required to qualify for FUO and which quantitative evaluation duration is required—3 or 7 days of hospitalization or 3 outpatient visits [1, 2]. Inflammation of unknown origin (IUO) has been described as a prolonged illness of >3 weeks with C-reactive protein levels >30 mg/L and/or erythrocyte sedimentation rates greater than age/2 in males (or [age + 10]/2 in females]) in the absence of temperatures ≥38.3°C (≥100.9°F) and uncertain diagnosis despite investigation after 3 days of hospitalization or 3 outpatient visits [3, 4].

**Table 1.** Fever of Unknown Origin: Definitions and Recommended Management

FUO Criteria and Recommendations	Petersdorf and Beeson (1961) [5]	Durack and Street (1991) [6]	de Kleijn et al^a^ (1997) [7]	Wright et al^a^ (2022) [1]	Delphi Panel^b^ (2023)
Temperature threshold	38.3°C (100.9°F)	38.3°C (100.9°F)	38.3°C (100.9°F)	38.0°C (100.4°F)	38.3°C (100.9°F)
Fever duration	≥3 wk	≥3 wk^c^	≥3 wk^d^	≥3 wk	≥3 wk
Epidemiology	Not considered	Not considered	Not specifically mentioned	Not specifically mentioned	Incorporate travel history, geographic residence, and prevalence of diseases
Evaluation setting	Inpatient	Inpatient/outpatient	Inpatient/outpatient	Inpatient/outpatient	Inpatient/outpatient
Evaluation duration	1 wk	3 d	NA	NA	NA
Recommendations					
Initial laboratory testing^a^	Not recommended	Not recommended^e^	Required^f^	Recommended^g^	Recommended^g^
Initial imaging^a^	None	None	Abdominal/pelvic CT, sinus plain radiographs of teeth indium 111 IgG scintigraphy	Abdominal US and posteroanterior lateral view chest plain radiography or chest/abdominal/pelvic CT	Chest/abdominal/pelvic CT preferred over abdominal US and posteroanterior lateral view chest plain radiography
Nuclear medicine imaging	None	None	Indium 111 IgG and gallium 67 scintigraphy imaging	^18^FDG-PET/CT imaging early in diagnostic evaluation	^18^FDG-PET/CT imaging after patients meet criteria for FUO and IUO
Molecular diagnostics	Not considered	Not considered	Not considered	Discussed but no formal recommendation	Considered if targeted pathogen testing negative
Genetic testing	Not considered	Not considered	Not considered	No recommendation	For unexplained relapsing/recurring, periodic fevers or suspected autoinflammatory diseases
Classification system of associated diseases	12 Groups: infectious, neoplastic diseases, collagen disease, pulmonary embolization, benign nonspecific pericarditis, sarcoidosis, hypersensitivity states, cranial arteritis, periodic disease, miscellaneous diseases, factitious fever, or no diagnosis	No criteria for disease subclassification was proposed; authors proposed dividing FUO into 4 distinct types: classic, nosocomial, neutropenic, and HIV related	7 Groups: infections, neoplasms, noninfectious inflammatory disorders, drug fever, factitious fever, miscellaneous disorders, or undiagnosed illness	5 Groups: infections, neoplasms, noninfectious inflammatory disorders, miscellaneous conditions, and undiagnosed illnesses	5 Groups: infections, neoplasms, noninfectious inflammatory disorders, miscellaneous conditions, and undiagnosed illnesses
Empirical therapy	Postpone or provide in selected conditions (eg, suspected endocarditis, tuberculosis, brucellosis, or tularemia)	Postpone except for fever in immune-compromised or neutropenic patients	No recommendation	Refrain except for the severely ill patient	Reserved for those in whom all other approaches have failed or those so seriously ill that therapy cannot be withheld for a further period of observation

Abbreviations: CT, computed tomography; ^18^FDG-PET/CT, 2-deoxy-2-[^18^F] fluoro-D-glucose positron emission tomography/computed tomography; FUO, fever of unknown origin; HIV, human immunodeficiency virus; IgG, immunoglobulin G; IUO, inflammation of unknown origin; NA, not available; US, ultrasonography.

^a^de Kleijn et al [7], Bleeker-Rovers et al [8], and Wright et al [1] recommended incorporating potential diagnostic clues from the history and physical examination when considering diagnostic testing and classified these initial laboratory and imaging tests as obligatory tests.

^b^The Delphi panel also included updated recommendations for the definition of IUO.

^c^HIV-associated FUO was defined as a fever >4 weeks in duration for outpatients or >3 days in duration in the hospital.

^d^Fever ≥38.3°C (≥100.9°F) on ≥3 occasions is also suggested.

^e^FUO diagnosis was uncertain after 3 days of “appropriate” laboratory or image investigations, including ≥2-day incubation of microbiologic cultures.

^f^Obligatory investigations occurred in 2 phases based on the presence or absence of potential diagnostic clues. Blood cultures incubating for >1 week were suggested.

^g^The standard minimum diagnostic protocol includes the following components: (1) laboratory tests (complete blood cell count including differential, comprehensive metabolic panel including calcium and liver function tests, erythrocyte sedimentation rate, C-reactive protein, and ferritin); (2) microbiology tests (blood cultures [≥3 sets with spacing; 5-day incubation], urinalysis and reflex to urine culture [≥1 set], and tuberculin skin test or interferon γ release assay); (3) imaging tests (must include both abdominal ultrasonography and posteroanterior lateral view chest plain radiography or chest/abdominal/pelvic CT).

Although FUO and IUO remain commonly used medical diagnoses, reaching a specific diagnosis as a cause is frequently challenging, with up to 51.0% of FUO and 40.0% of IUO cases remaining undiagnosed [1–3]. Despite a plethora of definitions based on expert opinion [5, 6, 8–10], there is a need to establish consensus on standardized diagnostic criteria, including the types of testing that may improve patient care and provide a solid foundation for research studies and comparisons [1–3]. Existing definitions fail to note factors such as country of residence, travel history, use of nuclear medicine techniques, molecular diagnostic methods, or empiric therapies (eg, antimicrobial agents or corticosteroids) [2–12]. Compared with studies performed ≥30 years ago, advancements in laboratory and imaging techniques and a better understanding of some febrile disorders’ pathogenic mechanisms offer opportunities for refinements [1, 12]. Since sufficient clinical evidence to address such questions is lacking, an appropriate approach would depend on experts in the field building recommendations based on clinical practices and available research, including FUO studies performed in the Netherlands [7–9, 11, 13, 14] and IUO studies in Belgium [3, 4, 10, 15–17].

One previous study investigated physicians’ attitudes toward evaluating hospitalized patients with FUO [9]. These researchers noted divergent opinions regarding possible causes and diagnostic approaches. Findings from that study do not generate specific recommendations for clinical care or research priorities and are not generalizable to other patient populations, clinical settings, and healthcare systems. Therefore, by gathering viewpoints from the perspectives of FUO and IUO expert clinicians and researchers worldwide, this study used a modified Delphi technique [18, 19] to develop a global consensus on recommendations for clinical management and research strategies that may improve care and develop research priorities based on knowledge gaps.

## METHODS

When evidence is insufficient, the Delphi technique is a potential tool for reaching consensus on clinical practice guidance and research needs [18, 19]. With an algorithm of temporally spaced sequential questionnaires, this technique gathers opinions on a given subject. It summarizes them for modification based on individual written responses instead of assembled participant groups. This technique helps professionals reach a consensus on subjects like FUO and IUO for which precise definitions or data-driven guidance might not be available. The essential elements include (1) anonymous responses from participating individuals, (2) interaction between participants following each round of input from questionnaire responses and controlled feedback to participants, and (3) statistical group response [18, 19].

We applied these techniques in compliance with the Conducting and Reporting of Delphi Studies (CREDES) standards [19]. We also adhered to the classic Delphi methodology for round 1, with an open-ended questionnaire that allowed expert panel members unbiased freedom in their responses to generate ideas or comments in subsequent survey rounds [19]. Although the term “modified Delphi” has no universally accepted definition, we chose to use it in this study because of our methodologic approach and the fact that the steering committee helped with group communication [19]. Finally, this study addresses classic FUO in the immunocompetent patient and excludes nosocomial, neutropenic, and human immunodeficiency virus–associated FUO [1, 6].

### Selection of Panelists

We sought to recruit a 50-member panel representing researchers and clinical experts who routinely participate in the care and research of patients with FUO or IUO, identified by peer-reviewed published articles within the past 20 years. The intended participants received an email containing a summary of the background, objective, design, and time needed for the study and a request for demographic information.

### Questionnaires

The questionnaires were administered anonymously by email using REDCap (Research Electronic Data Capture) over 4 main rounds (Supplementary Tables 1, 2, 3, and 4). Free-text options allowed panelists to express opinions in addition to structured questions. In subsequent rounds, the panelists ranked statements that provided the basis for the next round. Between rounds, data were analyzed and summarized by 2 researchers (W. F. W. and L. S.). Questionnaire refinements were discussed within the research group (all authors), with sufficiently ranked items brought into the next round. Requirements for consensus were that each statement had to meet ≥2 of the 3 criteria from the statistical analysis: (1) ≥80% of panelists agreed or disagreed; (2) a mean (average) score of ≥3.5 to 5.0 for agreement or ≤2.6 to 1.0 for disagreement; or) 3) a median score of ≥4.0 to 5.0 for agreement or ≤2.0 to 1.0 for disagreement.

### Institutional Review Board Review

The Johns Hopkins University School of Medicine institutional review board and Office of Research Assistance approved the study (IRB00326408).

### Patient Consent Statement

This study does not include factors necessitating patient consent, but each survey participant provided written informed consent.

### Statistical Analysis

The collected data were entered into Microsoft Excel and imported to the Stata version 17.0 software program for statistical analyses (StataCorp; 2022). Mean, median, and standard deviations were derived for key predictor variables and grouped into categories recommended by CREDES [19]. No formal methods were used to assess the risk of bias due to missing synthesis results.

## RESULTS

Twenty-six potential panelists (52.0% [n = 50]) registered and consented to participate. The modified 5-round Delphi technique is summarized in Supplementary Figure 1. The characteristics of panel members by round are presented in Table 2. Statements for each round are presented in Supplementary Tables 1, 2, 3, and 4.

**Table 2.** Characteristics of Survey Respondents by Round

Characteristic	Respondents, No. (%)			
	Round 1 (n = 23)^a^	Round 2 (n = 21)	Round 3 (n = 21)	Round 4 (n = 20)
Sex				
Male	17 (73.9)	15 (71.4)	15 (71.4)	14 (70.0)
Female	6 (26.1)	6 (28.6)	6 (28.6)	6 (30.0)
Age, y				
25–34	1 (4.3)	1 (4.8)	1 (4.8)	1 (5.0)
35–44	4 (17.4)	3 (14.3)	3 (14.3)	3 (15.0)
45–54	9 (39.1)	9 (42.9)	9 (42.9)	9 (45.0)
55–65	7 (30.4)	6 (28.6)	6 (28.6)	6 (30.0)
>65	2 (8.6)	2 (9.5)	2 (9.5)	1 (5.0)
Specialty				
Infectious diseases	10 (43.5)	10 (47.6)	10 (47.6)	9 (45.0)
Internal medicine	10 (43.5)	9 (42.9)	9 (42.9)	9 (45.0)
Pathology	1 (4.3)	1 (4.8)	1 (4.8)	1 (5.0)
Radiology	2 (8.7)	1 (4.8)	1 (4.8)	1 (5.0)
Hospital setting				
Academic	18 (78.3)	17 (80.9)	17 (80.9)	16 (80.0)
Community	5 (21.7)	4 (19.1)	4 (19.1)	4 (20.0)

^a^Panelists were from Belgium (n = 2), Canada (n = 1), England (n = 2), France (n = 3), India (n = 1), Italy (n = 1), Japan (n = 2), Netherlands (n = 3), North Macedonia (n = 1), Romania (n = 1), and the United States (n = 6).

### Questionnaire Round 1

A total of 23 registered panelists (88.5% [n = 26]) from 11 countries (Table 2) completed the round 1 survey; 95 statements were submitted from surveyed experts, ranging from 1 to 10 responses for each expert. Of these participants, 18 (78.3%) were academic-based clinicians and researchers, 5 (21.7%) practiced in a community-based hospital, and 6 (26.1%) were female. The mean physician practice was 21.8 years (range, 2–40 years; standard deviation, 9.92 years). Experts came from the following clinical specialties: 10 each (43.5%) from internal medicine and infectious diseases , 2 (8.7%) from radiology, and 1 (4.3%) from pathology.

### Questionnaire Round 2

In round 2, 21 (91.3% [n = 23]) of those who consented responded. A consensus was reached on most items (Supplementary Table 2 and Table 3), except for temperature thresholds, inflammatory criteria, the role of molecular diagnostics, testing for genetic disorders, and empirical treatment for stable patients. Eleven statements achieved strong consensus (3 of 3 criteria), 5 statements achieved moderate consensus (2 of 3 criteria), and 1 statement had moderate disagreement about the role of 2-deoxy-2-[^18^F] fluoro-D-glucose positron emission tomography/computed tomography (^18^FDG-PET/CT) (2 of 3 criteria).

**Table 3.** Round 2–3 Statements Reaching Consensus^a^

Strong consensus agreement (ie, met 3 of 3 criteria) Number of fever episodes (ie, >3) is an important component of the diagnostic criteria for FUO. A standard set of minimum diagnostic tests is an important component of the diagnostic criteria for FUO. If available, chest, abdominal, and pelvic CT (without chest radiography or abdominal ultrasonography) are sufficient imaging components for the minimum diagnostic criteria for FUO. The patient's immune status is an important component of the diagnostic criteria for FUO. If available, ^18^FDG-PET/CT is an important diagnostic test after a patient fulfills the FUO criteria with minimal diagnostic tests. Geographic residence is an important consideration in FUO diagnostic testing. Travel history is an important consideration in FUO diagnostic testing. Using a standardized set of FUO diagnostic disease categories (ie, infections, noninfection inflammatory disorders, oncology, miscellaneous, and undiagnosed) is important. Standard definitions for continuous and episodic fevers are important for FUO. A standard set of minimum diagnostic tests is an important component of the diagnostic criteria for IUO. Empirical therapy (eg, antimicrobials, corticosteroids, and anti-inflammatory agents) is important for unstable patients meeting FUO and IUO criteria and after appropriate investigations. Moderate consensus agreement (ie, met 2 of 3 criteria) The site of temperature measurement is an important component of the diagnostic criteria of FUO. The 3-week time-based criterion is an important component of the diagnostic criteria for FUO. A standard definition for relapsing fevers is important for FUO. The 3-week time-based criterion is an important component of the diagnostic criteria for IUO. The diagnostic approaches used in FUO can be applied to IUO. Moderate consensus disagreement (ie, met 2 of 3 criteria) A ^18^FDG-PET/CT scan result is an important component of the diagnostic criteria for FUO.

Abbreviations: CT, computed tomography; FUO, fever of unknown origin; IUO, inflammation of unknown origin; PET, positron emission tomography.

^a^The requirements for statements to reach consensus (ie, agreement or disagreement) was that each statement had to meet ≥2 of the 3 criteria from the statistical analysis: (1) >80% of panelists agreed or disagreed; (2) a mean (average) score of ≥3.5 to 5.0 for agreement or ≤2.6 to 1.0 for disagreement; and/or (3) a median score of ≥4.0 to 5.0 for agreement or ≤2.0 to 1.0 for disagreement.

### Questionnaire Round 3

Responses were received from 21 (80.8%) in round 3 (Table 3). Consensus was reached on nearly half (44.4% [n = 9]) of the items (Supplementary Table 3). Except for molecular diagnostics, genetic testing, and empirical therapy for stable patients. The temperature threshold of 38.0°C (100.4°F) was deemed more important than the earlier threshold of 38.3°C (100.9°F).

### Questionnaire Round 4

In round 4, 20 of 21 (95.2%) of those who consented responded to the open-ended style questionnaire (Supplementary Table 4). The primary purpose of round 4 was to provide context for a round 5 video meeting among the primary research panelists (all authors) on 9 of 10 topics that had not reached consensus in the previous rounds. The primary research panelists addressed quality clinical metrics for these conditions in a separate publication [20].

### Video Round 5 Delphi Panel Meeting

All 7 primary research panelists participated in 2 video meetings. During these meetings, 9 unresolved items from round 4 were discussed and condensed into a final list of recommendations (Table 4).

**Table 4.** Round 4 Questions and Results of Round 5 Video Consensus Recommendations From Study Researchers^a^

Question	Consensus Recommendation
What do you think is the best solution to incorporating molecular diagnostic methods (eg, universal 16S ribosomal RNA [rRNA] gene polymerase chain reaction [PCR] followed by Sanger sequencing, broad fungal sequencing using the D1/D2 region of the large subunit of the 28S rRNA gene, and internal transcribed spacer region [ITS]) into the evaluation of FUO and IUO patients?	Clinicians should consider these methods to be used later in the evaluation when infection is suspected based on history or PDCs, tissue biopsy results (eg, granulomatous pathology), and negative cultures.
2. What do you think is the best solution to incorporating ^18^FDG-PET/CT scans into evaluating FUO and IUO patients?	Clinicians should consider earlier use of ^18^FDG-PET/CT, after plain radiography or CT, particularly in the absence of PDCs.
3. How would you approach testing for genetic disorders for patients with FUO and IUO?	Clinicians should reserve genetic testing for cases associated with relapsing/recurring or periodic fevers and suspected autoinflammatory diseases later in the evaluation process.
4. What do you think is the best solution for medically managing symptoms for stable patients meeting FUO and IUO criteria and after appropriate investigations?	Clinicians should reserve symptomatic supportive therapy for patients presenting with alarm symptoms and diminished quality-of-life findings.
5. Do you feel it is necessary to objectify the fever to be able to fulfill the FUO criteria, should the temperature threshold for FUO be changed from 38.3C to 38.0C, and why?	Clinicians should objectively verify fevers in the setting of research studies and patients without symptoms or signs (eg, anemia or elevated inflammatory markers) of an underlying illness.
6. What do you think is the best anatomic site and method to obtain temperature measurements in the evaluation of FUO and IUO patients and why?	When fever is documented and reported, researchers and clinicians should clearly specify the cutoff temperature used in determining the presence of fever, the anatomic site at which temperatures are taken, and the instrument used to measure temperatures should be described.
7. What would you do to improve the current FUO and IUO diagnostic criteria?	Panelists agreed with improved definitions, considered adding major and minor criteria, and optimizing the minimal standard diagnostic evaluation. Additional panel considerations explored the use of precision medicine principles in these conditions.
8. What would you do to improve research efforts for FUO and IUO?	Panelists agreed with efforts to increase research funding, develop more multicenter studies, establish surveillance networks, recruit more members to the consensus group, and create an annual international consortium conference/workshop.
9. What do you think is the best solution to diagnostic disease categories (ie, infections, noninfection inflammatory disorders, oncology, miscellaneous, and undiagnosed) for FUO and IUO?	Researchers and clinicians should continue using the current 5 FUO disease category system.

Abbreviations: CT, computed tomography; FUO, fever of unknown origin; IUO, inflammation of unknown origin; PDCs, potential diagnostic clues; PET, positron emission tomography.

^a^The round 4 question regarding quality clinical metrics for these conditions were addressed by the primary research panelists in a separate publication [20].

## DISCUSSION

### Summary of Study

Using a modified Delphi approach, a diverse stakeholder group of subject matter experts reached a consensus on clinical and research priorities for FUO and IUO. There was notable agreement on 26 recommendations (Table 3). These findings fell into 5 themes: epidemiology, minimal diagnostic defining criteria, evaluation processes, classification of associated diseases, and empirical therapy (Table 5). There were mixed opinions around the issues of importance to the site of temperature measurement, the 3-week time-based criterion, the need for a standard definition for relapsing fevers, and the use of similar evaluation approaches for both conditions once a patient has been classified as having FUO or IUO (Table 4). To our knowledge, these findings constitute the first international consensus-driven clinical and research recommendations for these conditions.

**Table 5.** Summary of Full Panel Final Consensus Recommendations

Consensus recommendation 1	The panel recommends that clinicians incorporate geographic residence and the prevalence of diseases when considering diagnostic testing for FUO and IUO. Incorporating travel history is also recommended when considering potential diseases.
Consensus recommendation 2^a^	The panel recommends that the classification of FUO requires >3 wk of fever without explanation despite completing a set of minimal standard diagnostic tests in an immunocompetent patient. The panel recommends that if available, computed tomography of the chest, abdomen, and pelvis alone is preferred over chest plain radiography or abdominal ultrasonography as the imaging component for the minimum diagnostic criteria for FUO. The panel recommends classification of IUO as >3 wk of elevated inflammatory markers without fevers >38.3°C (>100.9°F) or explanation, despite completion of a set of minimal standard diagnostic tests in an immunocompetent patient.
Consensus recommendation 3	The panel recommends that, if available, ^18^FDG-PET/CT is an important early diagnostic test after a patient fulfills the FUO criteria with minimal diagnostic tests, particularly in the absence of potential diagnostic clues, but did not recommended incorporating ^18^FDG-PET/CT findings into the diagnostic criteria for FUO and IUO. The panel recommends that diagnostic approaches used in FUO can be applied to IUO.
Consensus recommendation 4	The panel recommends that clinicians and researchers use the standardized set of 5 diagnostic disease categories (ie, infections, noninfection inflammatory disorders, oncology, miscellaneous conditions, and undiagnosed illnesses) for FUO and IUO.
Consensus recommendation 5	The panel recommends that empirical therapeutic trials (eg, antimicrobials, corticosteroids, anti-inflammatory agents) be reserved for very few patients in whom all other approaches have failed or those so seriously ill that therapy cannot be withheld for a further period of observation.

Abbreviations: ^18^FDG-PET/CT, 2-deoxy-2-[^18^F] fluoro-D-glucose positron emission tomography/computed tomography; FUO, fever of unknown origin; IUO, inflammation of unknown origin.

^a^Minimal obligatory studies include complete blood cell count with differential, comprehensive metabolic panel with calcium and liver function tests, erythrocyte sedimentation rate, C-reactive protein, ferritin, thyroid-stimulating hormone, rheumatoid factor, antineutrophil cytoplasmic antibodies, and antinuclear antibodies. Microbiology studies include human immunodeficiency (HIV) 1/2 serology, urinalysis with microscopy, *Mycobacterium tuberculosis* skin test or whole-blood interferon-γ release assay, and blood cultures (3 sets). Imaging studies must include both abdominal ultrasonography and posteroanterior lateral view chest plain radiography or chest/abdominal/pelvic computed tomography.

^b^Criteria used to define an immunocompromised patient include neutropenia (neutrophil count <0.5 × 10^9^/L) for ≥1 week within 3 months before the start of the fever, receive immunosuppressive medications because of solid organ or hematologic stem cell transplant, known hypogammaglobulinemia, use of 10-mg prednisone or the equivalent for ≥2 weeks within 3 months before fever onset, uncontrolled virus HIV infection (CD4 cell count <200/mL), or receipt of biologic therapies (eg, anti–tumor necrosis factor or monoclonal antibodies).

### Theme 1: Epidemiology—Consensus Recommendation 1

The panel recommends that clinicians incorporate geographic residence and the prevalence of diseases when considering diagnostic testing for FUO and IUO. Incorporating travel history is also recommended when considering potential diseases.

Analyses by geographic region, based on the World Health Organization and World Bank Country classification systems, among several meta-analyses, reported significant geographic variations in the diagnostic outcomes [2, 21–24]. Patients from Southeastern Asia regions and lower-middle-income countries were more likely than those from European and high-income countries to have an infectious disease rather than a noninfectious inflammatory disorder [22–25]. While some geographic regions lacked data for comparison, such as Africa and North and South America, the practical implications from these meta-analyses suggested that physicians worldwide should incorporate geographic disease prevalence when evaluating these patients. No study has evaluated the predictive value of obtaining a travel history and subsequent outcomes.

### Theme 2: Minimal Defining Criteria—Consensus Recommendation 2

The panel recommends that the classification of FUO requires ≥3 weeks of fever without explanation after completion of a set of minimal standard diagnostic tests in an immunocompetent patient (Table 5). The panel recommends that, if available, computed tomography of the chest, abdomen, and pelvis alone should be preferred over chest plain radiography or abdominal ultrasonography as the imaging component for the minimum diagnostic criteria for FUO. For IUO, the panel recommends ≥3 weeks of elevated inflammatory markers without fever ≥38.3°C (≥100.9°F) or explanation after completion of a set of minimal standard diagnostic tests in an immunocompetent patient.

Using the 3-week duration for FUO [1] and IUO [3] avoids self-limiting infectious diseases (eg, viral infections) and ensures that a persistent febrile or inflammatory condition exists. While the duration is somewhat arbitrary, some have proposed a shorter duration of 2 weeks to avoid delay [1]. Regardless, experts believed that 3 weeks remains a crucial component of the definitions, as it is essential to exclude self-limiting infections [1]. No study to our knowledge has directly compared outcomes based on the duration of fever (ie, 2 vs 3 weeks), which is an identified research gap.

Although several meta-analyses have been published [2, 14, 22–25], the optimal FUO and IUO criteria and evaluation methods remain unanswered by clinical trial evidence. Panelists from this study proposed that clinicians and researchers use minimal standard diagnostic testing in an immunocompetent patient for FUO and IUO criteria, as this would improve comparability among future research studies [1, 3]. Including immunocompromised patients in the standard definition would alter diagnostic method comparisons, causes of fever, and treatment advances [1]. Finally, the panel agreed that, for IUO, no changes were required for inflammatory marker values [3, 4].

### Theme 3: Evaluation—Consensus Recommendation 3

The panel recommends that, if available, ^18^FDG-PET/CT is an essential early diagnostic test after a patient fulfills the FUO criteria following the performance of minimal diagnostic tests, particularly in the absence of potential diagnostic clues, but that it should not be incorporated into the diagnostic criteria for FUO and IUO. The panel recommends that diagnostic approaches used in FUO can be applied to IUO.

The first consensus guideline for the appropriate use criteria of nuclear medicine for adults in FUO and IUO was published by the Society of Nuclear Medicine and Molecular Imaging [26]. Pooled data from this guideline and several other meta-analyses [26–32] demonstrate impressive diagnostic contributions with yields of 76%–83% for ^18^FDG PET and 84%–98% for ^18^FDG-PET/CT. Notably, patients with either infection or cancer benefited more from ^18^FDG-PET/CT than those with noninfectious inflammatory disorders [27]. In addition, negative ^18^FDG-PET/CT was correlated with spontaneous remissions that were approximately 6 times higher (relative risk [RR], 5.6 [95% confidence interval, 3.4–9.2]; *P* < .001) than in patients with positive results [30]. These findings suggest that ^18^FDG-PET/CT imaging should be performed earlier if remaining in limbo (ie, after a patient fulfills defining criteria) and also could provide prognostic information if negative [26, 27, 33, 34].

Gallium 67 scintigraphy should be reserved for situations where ^18^FDG-PET/CT is unavailable [26]. Another alternative, labeled leukocyte scintigraphy, should be reserved for those situations in which ^18^FDG-PET/CT is not available, and there is a high index of suspicion for infection as the cause of the fever [26].

### Theme 4: Classification of Diseases—Consensus Recommendation 4

The panel recommends that clinicians and researchers organize diseases associated with FUO and IUO using 5 diagnostic disease categories (ie, infections, noninfection inflammatory disorders, oncology, miscellaneous conditions, and undiagnosed illnesses). There has yet to be universal agreement regarding a uniform set of FUO disease classifications; studies over the past several decades have grouped diagnoses into the above categories [1, 14].

Some investigators (W.F.W. and P.G.A.) who were part of the primary research panel have proposed updating the classification of FUO subcategories based on a small but statistically significant difference between investigator-determined and *International Classification of Diseases, Tenth Revision* (*ICD-10*)–adjusted diagnoses [14]. The proportion of patients with a difference between the investigator and *ICD-10*–adjusted noninfectious inflammatory disorder category was 1.2% (95% confidence interval, .005–.021; *P* < .001), and the proportion was similar for the miscellaneous category at 1.5% (.007–.025; *P* < .001) [14]. The full panel did not endorse this approach, given the minor differences, unless further research justifies such a change.

### Theme 5: Empirical Therapy—Consensus Recommendation 5

The panel recommends that empiric therapeutic trials (eg, antimicrobials, corticosteroids, or anti-inflammatory agents) be reserved for select patients whose diagnostic evaluation has been exhausted and when a clinician believes the illness warrants such therapies instead of observation.

Studies suggest that cases of FUO that defy precise diagnosis after intensive investigation and prolonged observation usually carry a favorable prognosis [1, 13, 35]. Experts in the field have emphasized that therapeutic trials should be postponed until thoughtful diagnostic testing has been exhausted to avoid the possible negative effects upon establishing a final diagnosis or adverse drug reactions [1]. To date, no study has evaluated the predictive value of withholding or initiating empirical treatments and subsequent outcomes.

### Additional Panel Recommendations

Recommendations considered important by this study's participants but fall short of reaching panel consensus include incorporating molecular and genetic testing, objective verification of fevers, anatomic site of temperature measurement, and research goals (Table 4). For example, the recommendation to verify fever received much lower attention in this study since these items should be readily available in routine clinical care or are perceived as making little difference in the patient’s care or outcome, particularly for patients with elevated inflammatory markers or anemia [14]. However, for the subset of patients with normal inflammatory marker levels or without anemia, we would advocate objectively verifying fevers before subjecting the patient to an extensive clinical evaluation or research protocol and exclude factitious fevers [1, 14]. When fever is documented and reported, we and others [36] recommend that researchers and clinicians also indicate the threshold used, the anatomic site where temperatures are taken, and the specific instrument used to measure temperature.

While panelists favored lowering the traditional threshold of 38.3°C (100.9°F) for FUO to 38.0°C (100.4°F), this did not reach a consensus. Reasons for lowering the threshold are that (1) 38.0°C is considered the standard upper level of normal and (2) investigators have reported mean body temperatures in men and women, after adjustment for age, height, and weight, have decreased monotonically by 0.03°C per birth decade since the 1890s [1, 37]. Therefore, lowering the temperature threshold for FUO might improve sensitivity [1]. A change in temperature threshold would affect the definition and spectrum of disorders associated with IUO. Finally, while the panelists proposed ≥3 fever episodes as criteria, there are no precise data to define this point.

Data for the routine use of molecular methods in cases of FUO and IUO are sparse [38, 39]. Currently, DNA- or RNA-based molecular testing (single or multiplex) should be targeted to possible diagnoses based on a differential diagnosis. Next-generation sequencing may be considered if targeted testing is unfruitful for suspected infection but remains unexplained despite testing. Genetic testing data for FUO or IUO are also limited [40, 41]. With our current knowledge base, these methods should be reserved for unexplained relapsing/recurring or periodic fevers or suspected autoinflammatory diseases.

### Implications for Research

Evaluation of these recommendations would require comparing criteria and evaluation methods using receiver operating characteristic curve sensitivity versus specificity across a range of geographic locations and clinical outcomes. Developing and evaluating clinical end points for these conditions would be expected to be complex and time consuming and to involve numerous stakeholders. Recommendations must also be assessed for feasibility in routine clinical practice with minimal unintended consequences, such as increasing clinician and patient burdens, implementation costs, or clinical workflow changes. Therefore, a trial design of quality indicators comparing management strategies using desirability of outcomes rankings end points could provide the pragmatic and patient-centered information clinicians need to make informed decisions about patient care that could be developed through a survey of clinician-scientists and clinical trialists using case vignettes [20, 42, 43].

### Limitations

This study has some limitations. First, these recommendations represent the consensus of a limited number of subject-area experts. Moreover, stakeholders were primarily men from academia. Greater representation of more male and female experts from a wider array of public and private sector entities across geographically broader healthcare systems would have been beneficial, along with incorporation of patient input. For example, including patients, private practice clinicians, other medical specialists (eg, rheumatology and oncology), government and legislative officials, representatives from public health, and medical product developers would have added to the diversity of perspectives. Second, the language of the questions and statements generated could be interpreted differently without context. The researchers’ peer person-to-person dialogue played a critical role in clarifying the language throughout the process. Still, some ambiguity could have remained due to the limited time frame. This was addressed, in part, by sharing a draft of the prioritized questions with all the primary researcher participants in this study for review and final approval. Finally, some studies have found potential cost savings when performing ^18^FDG-PET/CT or advanced molecular methods, particularly early in the evaluation of FUO and IUO [27, 38, 44]. However, this Delphi panel did not address this issue, which merits further study and discussion.

## Conclusions

The International Fever and Inflammation of Unknown Origin Research Working Group developed the first consensus-based set of clinical and research recommendations for patients with FUO or IUO. Given the lack of a universally agreed-upon definition and guideline for care, recommendations from this study may currently serve in this role, as they are based on a broad consensus from international experts, recognizing that future changes brought by discovery and practice changes may require periodic revisiting. Investigators, clinicians, professional societies, and funding agencies should use these recommendations to inform future research and funding priorities to improve clinical care and reduce undiagnosed illnesses.

## Supplementary Data

Supplementary materials are available at *Open Forum Infectious Diseases* online. Consisting of data provided by the authors to benefit the reader, the posted materials are not copyedited and are the sole responsibility of the authors, so questions or comments should be addressed to the corresponding author.

## Supplementary Material

ofae298_Supplementary_Data
