# Supplementary material for: Recommendations for Updating Fever and Inflammation of Unknown Origin From a Modified Delphi Consensus Panel
Source: Open Forum Infect Dis. 2024 Jun 10;11(7):ofae298. doi: 10.1093/ofid/ofae298 (PMC11222709; doi:10.1093/ofid/ofae298)
Supplement: ofae298_Supplementary_Data [file ofae298_supplementary_data.docx]

**Supplementary data**

**eFigure 1.** Delphi Survey Flowchart

**Pre-Delphi Recruitment.** 50 Experts recruited; 26 respondents consented to participate

↓

**Round 1. Delphi generation.** Single open-ended question; 23 respondents completed the survey

↓

**Round 2. Item reduction.** Rank the top 26 items from the previous round; 21 respondents completed the survey

↓

**Round 3. Item reduction.** Rank 9 items from the previous round; 21 respondents completed the survey

↓

**Round 4. Item reduction.** Rank 9 items from the previous round; 20 respondents completed the survey

↓

**Round 5. Final ranking of unresolved items.** Seven research panelists from this study participated in two video meetings to rank 9 items from the previous round

**eTable 1. Questionnaire Round 1**

**Study title: A Delphi consensus study to identify research priorities for the Fever and Inflammation of Unknown Origin Syndrome**

The first round of this Delphi will ask you a question – “What are the current research priorities for the FUO and IUO syndrome?”

We ask that you consider features that could be considered for inclusion in the diagnostic criteria, including features on history, physical examination and investigation methods. Also consider both resource-limited and high income settings as well as hospital and office-based settings.

There are ten (10) spaces for you to detail your answers. You can complete as many or as few of these spaces as you wish. Please be as detailed in your response as possible.

**Delphi Round 1**

Please list your answers to the following question. You can list as many answers as you wish and they do not have to be in any particular order.

**Question:** **What are the current research priorities for the FUO and IUO syndrome?**

**1.**

**2.**

**3.**

**4.**

**5.**

**6.**

**7.**

**8**

**9.**

**10.**

**eTable 2. Delphi Questionnaire Round 2.**

For each of the statements below, select the response that best characterizes how you feel about the statement, where: 1 = Strongly Disagree, 2 = Disagree, 3 = Neither Agree Nor Disagree, 4 = Agree, and 5 = Strongly Agree.

|  | Strongly Disagree | Disagree | Neither Agree Nor Disagree | Agree | Strongly Agree |
| --- | --- | --- | --- | --- | --- |
| 1. The temperature threshold of 38.3 C is an important component of the criteria for FUO. | 1 | 2 | 3 | 4 | 5 |
| 2. The temperature threshold of 38.0 C is an important component of the criteria for FUO. | 1 | 2 | 3 | 4 | 5 |
| 3. The site of temperature measurement is an important component for the diagnostic criteria of FUO. | 1 | 2 | 3 | 4 | 5 |
| 4. The number of fever episodes (e.g., greater than 3) is an important component of the diagnostic criteria for FUO. | 1 | 2 | 3 | 4 | 5 |
| 5. The 3-week time-based criterion is an important component of the diagnostic criteria for FUO. | 1 | 2 | 3 | 4 | 5 |
| 6. A standard set of minimum diagnostic tests is an important component of the diagnostic criteria for FUO. | 1 | 2 | 3 | 4 | 5 |
| 7. The combination of a chest x-ray and abdominal ultrasound (without CT-scan) are sufficient imaging components for the minimum diagnostic criteria for FUO. | 1 | 2 | 3 | 4 | 5 |
| 8. If available, a chest, abdomen, and pelvic CT-scan (without chest x-ray or abdominal ultrasound) are sufficient imaging components for the minimum diagnostic criteria for FUO. | 1 | 2 | 3 | 4 | 5 |
| 9. The patient’s immune status is an important component of the diagnostic criteria for FUO. | 1 | 2 | 3 | 4 | 5 |
| 10. A PET/CT scan result is an important component of the diagnostic criteria for FUO. | 1 | 2 | 3 | 4 | 5 |
| 11. If available, a PET/CT scan result is an important diagnostic test after a patient fulfills the FUO criteria with minimal diagnostic tests. | 1 | 2 | 3 | 4 | 5 |
| 12. If available, molecular diagnostic methods (e.g., universal 16S ribosomal RNA [rRNA] gene polymerase chain reaction [PCR] followed by Sanger sequencing, broad fungal sequencing using the D1/D2 region of the large subunit of the 28S rRNA gene, and internal transcribed spacer region [ITS]) are important early phase after a patient fulfills the FUO criteria. | 1 | 2 | 3 | 4 | 5 |
| 13. After a patient fulfills FUO criteria with standard diagnostic tests and exclusion of infections, testing for genetic disorders is important. | 1 | 2 | 3 | 4 | 5 |
| 14. Geographic residence is an important consideration in FUO diagnostic testing. | 1 | 2 | 3 | 4 | 5 |
| 15. Travel history is an important consideration in FUO diagnostic testing. | 1 | 2 | 3 | 4 | 5 |
| 16. Using a standardized set of FUO diagnostic disease categories (e.g., infections, non-infection inflammatory disorders, oncology, miscellaneous, and undiagnosed) is important. | 1 | 2 | 3 | 4 | 5 |
| 17. Standard definitions for continuous and episodic fevers are important for FUO. | 1 | 2 | 3 | 4 | 5 |
| 18. A standard definition for relapsing fevers are important for FUO. | 1 | 2 | 3 | 4 | 5 |
| 19. FUO and inflammation of unknown origin (IUO) are the same clinical syndrome. | 1 | 2 | 3 | 4 | 5 |
| 20. Raised inflammatory markers (C-reactive protein [CRP] >30 mg/L, erythrocyte sedimentation rate [ESR] > age/2 in males or (age+10)/2 in females, and serum amyloid A [SAA] >10mg/L) on more than 3 occasions is an important component of the criteria for IUO. | 1 | 2 | 3 | 4 | 5 |
| 21. The 3-week time-based criterion is an important component of the diagnostic criteria for IUO. | 1 | 2 | 3 | 4 | 5 |
| 22. A standard set of minimum diagnostic tests is an important component of the diagnostic criteria for IUO. | 1 | 2 | 3 | 4 | 5 |
| 23. After a patient fulfills IUO criteria with standard diagnostic tests and exclusion of infections, testing for genetic disorders is important. | 1 | 2 | 3 | 4 | 5 |
| 24. The diagnostic approaches used in FUO can be applied to IUO. | 1 | 2 | 3 | 4 | 5 |
| 25. Empirical therapy (e.g., antimicrobials, corticosteroids, anti-inflammatory agents) are important for stable patients meeting FUO and IUO criteria and after appropriate investigations. | 1 | 2 | 3 | 4 | 5 |
| 26. Empirical therapy (e.g., antimicrobials, corticosteroids, anti-inflammatory agents) are important for unstable patients meeting FUO and IUO criteria and after appropriate investigations. | 1 | 2 | 3 | 4 | 5 |

**eTable 3. Questionnaire Round 3**

For each of the statements below, select the response that best characterizes how you feel about the statement, where: 1 = Very important, 2 = Important, 3 = Neither important nor not important, 4 = Not important, and 5 = Unimportant.

| **Statement** | **Overall group response**  **(Median score)** | **Your Round 3 Response** | | | | |
| --- | --- | --- | --- | --- | --- | --- |
|  |  | **Very important** | **Important** | **Neither important nor not important** | **Not important** | **Unimportant** |
| 1. The temperature threshold of 38.3 C is an important component of the criteria for FUO. |  | 1 | 2 | 3 | 4 | 5 |
| 2. The temperature threshold of 38.0 C is an important component of the criteria for FUO. |  | 1 | 2 | 3 | 4 | 5 |
| 3. The combination of a chest x-ray and abdominal ultrasound (without CT-scan) are sufficient imaging components for the minimum diagnostic criteria for FUO. |  | 1 | 2 | 3 | 4 | 5 |
| 4. If available, molecular diagnostic methods (e.g., universal 16S ribosomal RNA [rRNA] gene polymerase chain reaction [PCR] followed by Sanger sequencing, broad fungal sequencing using the D1/D2 region of the large subunit of the 28S rRNA gene, and internal transcribed spacer region [ITS]) are important early phase after a patient fulfills the FUO criteria. |  | 1 | 2 | 3 | 4 | 5 |
| 5. After a patient fulfills FUO criteria with standard diagnostic tests and exclusion of infections, testing for genetic disorders is important. |  | 1 | 2 | 3 | 4 | 5 |
| 6. FUO and inflammation of unknown origin (IUO) are the same clinical syndrome. |  | 1 | 2 | 3 | 4 | 5 |
| 7. Raised inflammatory markers (C-reactive protein [CRP] >30 mg/L, erythrocyte sedimentation rate [ESR] > age/2 in males or (age+10)/2 in females, and serum amyloid A [SAA] >10mg/L) on more than 3 occasions is an important component of the criteria for IUO. |  | 1 | 2 | 3 | 4 | 5 |
| 8. After a patient fulfills IUO criteria with standard diagnostic tests and exclusion of infections, testing for genetic disorders is important. |  | 1 | 2 | 3 | 4 | 5 |
| 9. Empirical therapy (e.g., antimicrobials, corticosteroids, anti-inflammatory agents) are important for stable patients meeting FUO and IUO criteria and after appropriate investigations. |  | 1 | 2 | 3 | 4 | 5 |

| **eTable 4. Questionnaire Round 4** | |
| --- | --- |
| Question | Your answer |
| 1. What do you think is the best solution to incorporating molecular diagnostic methods (e.g., universal 16S ribosomal RNA [rRNA] gene polymerase chain reaction [PCR] followed by Sanger sequencing, broad fungal sequencing using the D1/D2 region of the large subunit of the 28S rRNA gene, and internal transcribed spacer region [ITS]) into the evaluation of FUO and IUO patients? |  |
| 1. What do you think is the best solution to incorporating PET/CT scans into the evaluation of FUO and IUO patients? |  |
| 1. How would you approach testing for genetic disorders for FUO and IUO patients? |  |
| 1. What do you think is the best solution for medically managing symptoms for stable patients meeting FUO and IUO criteria and after appropriate investigations? |  |
| 1. Do you feel it is necessary to objectify the fever to be able to fulfill the FUO criteria and should the temperature threshold for FUO be changed from 38.3C to 38.0C and why? |  |
| 1. If you could choose a quality clinical metric for FUO and IUO outcomes, what would you choose and why? |  |
| 1. What do you think is the best anatomical site and method to obtain temperature measurements in the evaluation of FUO and IUO patients and why? |  |
| 1. What would you do to improve the current FUO and IUO diagnostic criteria? |  |
| 1. What would you do to improve research efforts for FUO and IUO? |  |
| 1. What do you think is the best solution to diagnostic disease categories (e.g., infections, non-infection inflammatory disorders, oncology, miscellaneous, and undiagnosed) for FUO and IUO? |  |
